# Supplementary material for: Optimization of the Electro-Peroxone Process for Micropollutant Abatement Using Chemical Kinetic Approaches
Source: Molecules. 2019 Jul 20;24(14):2638. doi: 10.3390/molecules24142638 (PMC6680746; doi:10.3390/molecules24142638)
Supplement: Supplementary file 1 [file molecules-24-02638-s001.pdf]

molecules

Supporting Information

Optimization of the Electro-Peroxone Process for Micropollutant Abatement Using Chemical Kinetic Approaches

Huijiao Wang [1,2], Lu Su [3], Shuai Zhu [4], Wei Zhu [5], Xia Han [5], Yi Cheng [1], Gang Yu [2] and Yujue Wang [2,*]

1 Department of Chemical Engineering, Tsinghua University, Beijing 100084, China
2 School of Environment, Beijing Key Laboratory for Emerging Organic Contaminants Control, State Key Joint Laboratory of Environmental Simulation and Pollution Control, Tsinghua University, Beijing 100084, China
3 Hangzhou Jinhong Real Estate Co., Ltd., Hangzhou 310000, China
4 Beijing Guohuan Tsinghua Environmental Engineering Design & Research Institute Co., Ltd, Beijing 100084, China
5 Sinopec Energy and Environmental Engineering Co., Ltd., Wuhan 430070, China
* Correspondence: wangyujue@tsinghua.edu.cn; Tel.: +86-10-62772914

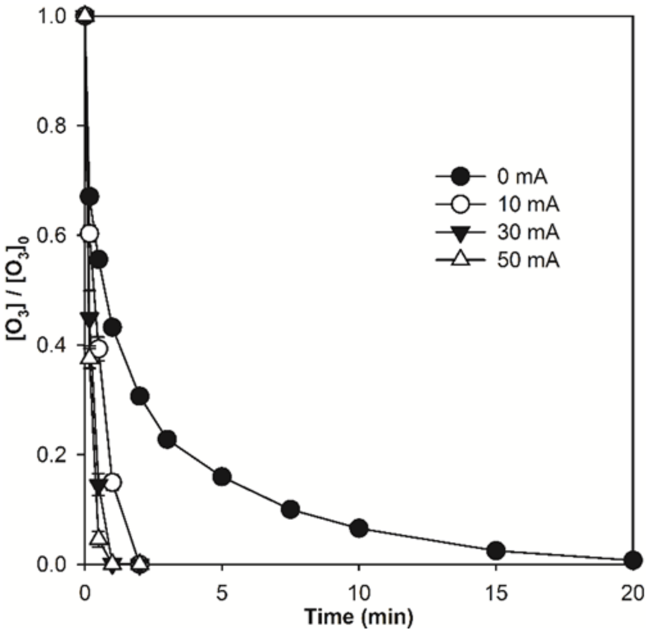

Figure S1. O_3 decay during E-peroxone treatment of surface water at different currents (0 mA (ozonation), 10 mA, 30 mA, and 50 mA). (Reaction conditions: each micropollutant concentration ~150 μg/L, and specific ozone dose = 1.5 mg O_3/mg dissolved organic carbon (DOC)).

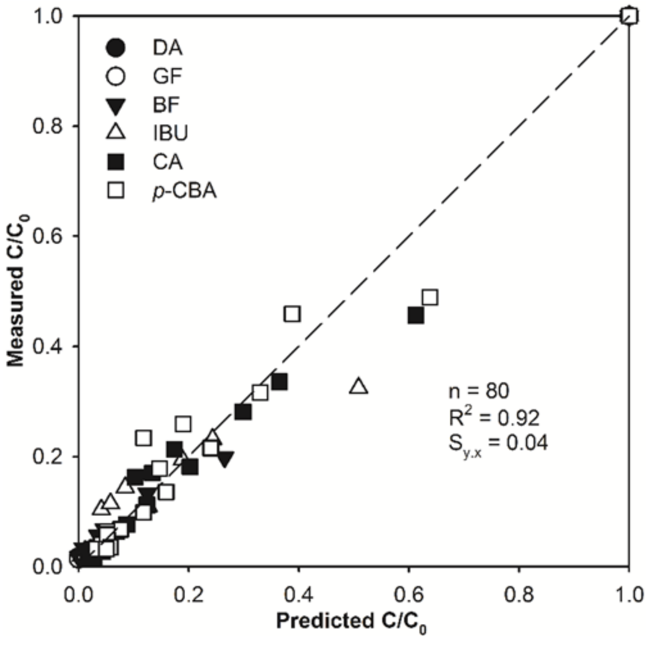

Figure S2. Correlation analysis between experimentally measured and model predicted micropollutant abatement by the E-peroxone process at different currents (0 mA (ozonation), 10 mA, 30 mA, and 50 mA). n is the number of data points, R^2 is the correlation coefficient, and $S_{y,x}$ is the standard deviation of the linear regression with an equation y=x. (Reaction conditions: each micropollutant concentration ~150 μg/L, and specific ozone dose = 1.5 mg O_3/mg dissolved organic carbon (DOC)).

To get further into the degradation pathway, the contribution of directly electrolysis (f_E), O_3 oxidation (f_{O3}), and •OH oxidation ($f_{•OH}$) to micropollutant abatement were calculated at 2 min of E-peroxone process when O_3 was completely depleted according to Equations (S1)–(S3) and presented in Figure. S3.

$$f_E = \frac{k_E t}{k_{•OH} \int[•OH]dt + k_{O3} \int[O_3]dt + k_E t} \quad (S1)$$

$$f_{O_3} = \frac{k_{O3} \int[O_3]dt}{k_{•OH} \int[•OH]dt + k_{O3} \int[O_3]dt + k_E t} \quad (S2)$$

$$f_{•OH} = \frac{k_{•OH} \int[•OH]dt}{k_{•OH} \int[•OH]dt + k_{O3} \int[O_3]dt + k_E t} \quad (S3)$$

where k_E is the pseudo-first order rate constant during electrolysis, and k_{O3} and $k_{•OH}$ are the second order rate constants with O_3 and •OH, respectively.

As shown in Figure. S3, direct electrolysis played negligible role on the removal of all tested micropollutants, with f_E less than 0.2%. This is mainly due to its rather slow abatement kinetics limited by mass transfer of pollutants to the electrode surface [1,2]. As a consequence, O_3 and •OH oxidation dominated micropollutant abatements during the E-peroxone process. For DA and GF with $k_{O3} > 10^4$ M^{-1} s^{-1}, they were mainly abated via O_3 oxidation ($f_{O3} > 93\%$). In comparison, BF, IBU, CA, and *p*-CBA have relatively low reactivity with O_3 ($k_{O3} < 590$ M^{-1} s^{-1}), and thus •OH oxidation dominated their abatements during the E-peroxone process ($f_{•OH} \geq 81\%$).

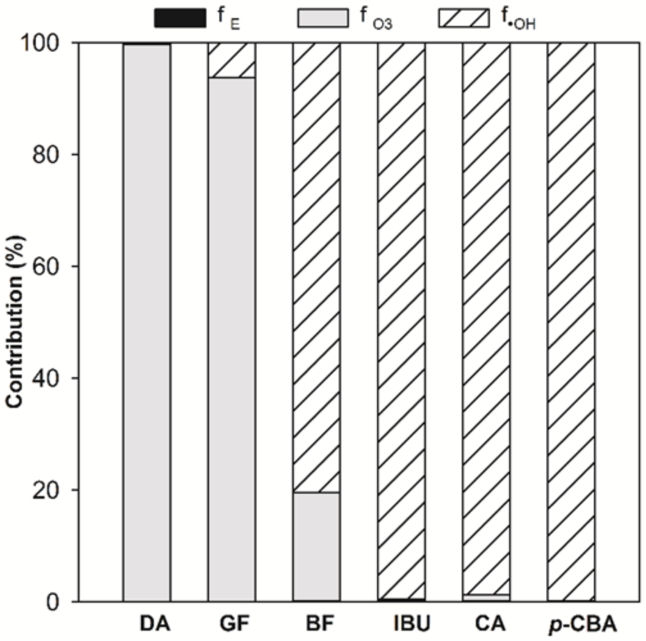

Figure S3. Contribution of electrolysis (f_E), O_3 oxidation (f_{O3}), and •OH oxidation ($f_{•OH}$) to micropollutant abatement during the E-peroxone treatment of surface water. (Reaction conditions: current = 30 mA, each micropollutant concentration ~150 μg/L, and specific ozone dose = 1.5 mg O_3/mg dissolved organic carbon (DOC)).

The current efficiency (CE) of H_2O_2 electro-generation was calculated according to Equation (S4), where n is the number of electrons consumed for converting O_2 to H_2O_2 (2 electrons), F is the Faraday constant (96,486 C/mol), C_{H2O2} is the concentration of H_2O_2 electro-generated (M), V is the solution volume (L), I is the current (A), and t is the reaction time (s).

$$CE(\%) = \frac{nFC_{H2O2}V}{\int_0^t Idt} \times 100 \quad (S4)$$

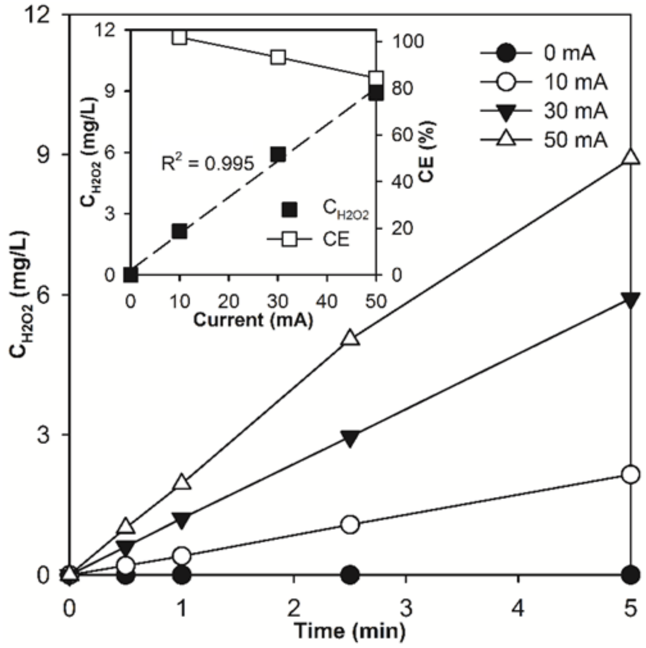

Figure S4. Electro-generation of H_2O_2 from cathodic O_2 reduction in the surface water at different currents. The inset plot shows H_2O_2 concentration at 5 min and H_2O_2 electro-generation current efficiency (CE) as a function of applied currents. (Reaction conditions: volume = 250 mL, current = 30 mA, Pt anode = 2 cm × 2 cm, carbon-PTFE cathode = 2 cm × 5 cm).

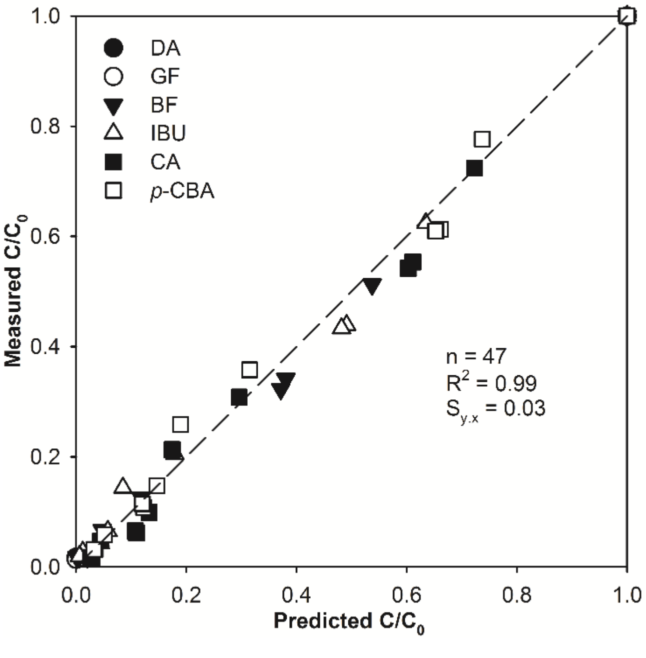

Figure S5. Correlation analysis between experimentally measured and model predicted micropollutant abatement by the E-peroxone process at different specific ozone dose (0.5, 1.0, and 1.5 mg O_3/mg dissolved organic carbon (DOC)). n is the number of data points, R^2 is the correlation coefficient, and $S_{y,x}$ is the standard deviation of the linear regression with an equation y=x. (Reaction conditions: each micropollutant concentration ~150 μg/L, and current = 30 mA).

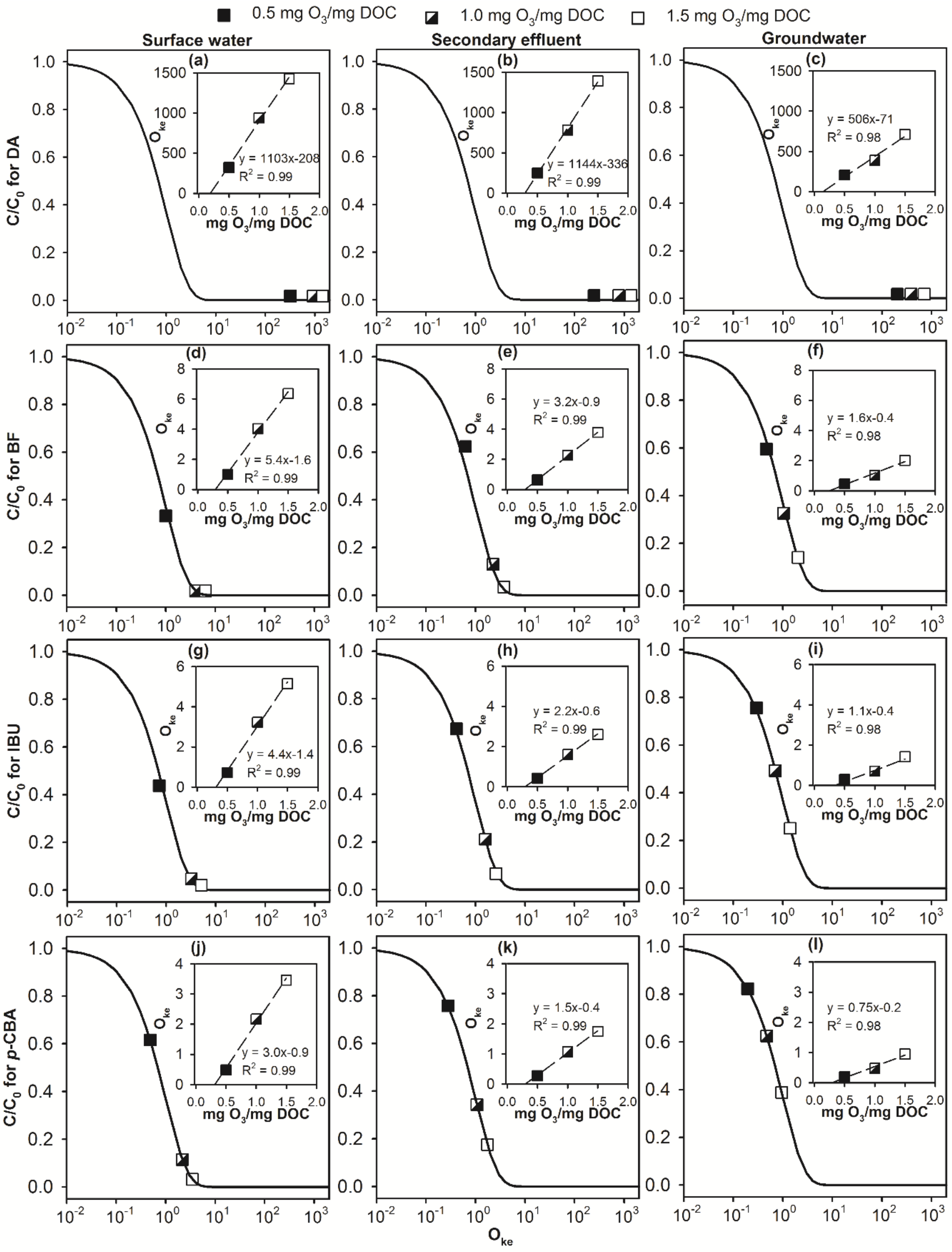

Figure S6. Abatement efficiency of (**a**–**c**) diclofenac (DA), (**d**–**f**) bezafibrate (BF), (g–i) ibuprofen (IBU), and (**j**–**l**) para-chlorobenzoic acid (*p*-CBA) as a function of their O_{ke} values during the E-peroxone treatment of surface water (left column), secondary effluent (middle column), and groundwater (right column) with varying specific ozone (O_3) doses. The symbols in the plot represent experimentally measured results; the solid lines are model simulation using Equation (3). The inset plot shows linear regression between specific O_3 dose and the O_{ke} value observed for selected micropollutant during the E-peroxone process. (Reaction conditions: current = 30 mA, and each micropollutant concentration ~150 μg/L).

Table 1. Model compounds used in this study and their second-order reaction rate constants with O_3 and •OH.

Compound	k_{O3} (M^{-1} s^{-1})	$k_{•OH}$ (M^{-1} s^{-1})
Diclofenac (DA)	6.8×10^5 [3]	7.5×10^9 [3]
Gemfibrozil (GF)	5×10^4 [4]	10×10^9 [4]
Bezafibrate (BF)	590 [3]	7.4×10^9 [3]
Ibuprofen (IBU)	9.6 [3]	7.4×10^9 [3]
Clofibric acid (CA)	< 20 [5]	5.2×10^9 [5]
para-chlorobenzoic (*p*-CBA)	≤ 0.15 [6]	5×10^9 [6]

Table 2. Main water quality parameters of the surface water, secondary effluent, and groundwater used in this study.

Parameter	Surface water	Secondary effluent	Groundwater
pH	8.2	8.0	8.1
DOC (mg/L)	4.0	6.2	1.2
HCO_3^- (mg/L)	131	196	295
CO_3^{2-} (mg/L)	2	3	6
Alkalinity (mg/L as $CaCO_3$)	110	165	248
Conductivity (μS/cm)	272	857	683
TDS (mg/L)	102	522	371

Table 3. Operational parameters for UPLC/MS-MS analysis and the limits of detection (LOD) and quantification (LOQ) of the tested micropollutants.

Compound	Precursor ion (*m*/*z*)	Product ion (*m*/*z*)	Declustering potential (V)	Collision energy (eV)	LOD (μg/L)	LOQ (μg/L)
DA	293.9	250.0	–15.0	–12.0	0.8	2.6
GF	249.3	121.0	–25.0	–18.0	0.6	2.1
BF	360.0	274.0	–40.0	–16.0	0.8	2.6
IBU	204.9	161.0	–24.0	–16.7	0.9	2.9
CA	212.8	126.9	–15.0	–20.0	0.8	2.6
p-CBA	154.9	111.0	–35.0	–18.0	1.4	4.7

References

1. Yao, W.; Wang, X.; Yang, H.; Yu, G.; Deng, S.; Huang, J.; Wang, B.; Wang, Y. Removal of pharmaceuticals from secondary effluents by an electro-peroxone process. *Water Res.* **2016**, *88*, 826–835. doi:10.1016/j.watres.2015.11.024.
2. Chaplin, B.P. Critical review of electrochemical advanced oxidation processes for water treatment applications. *Environ. Sci. Process. Impacts* **2014**, *16*, 1182–1203. doi:10.1039/C3EM00679D.
3. Huber, M.M.; Canonica, S.; Park, G.-Y.; von Gunten, U. Oxidation of pharmaceuticals during ozonation and advanced oxidation processes. *Environ. Sci. Technol.* **2003**, *37*, 1016–1024, doi:10.1021/es025896h.
4. Von Sonntag, C.; von Gunten, U. *Chemistry of Ozone in Water and Wastewater Treatment: From Basic Principles to Applications*; IWA Publishing: London, UK, 2012.
5. Huber, M.M.; Gobel, A.; Joss, A.; Hermann, N.; Loffler, D.; McArdell, C.S.; Ried, A.; Siegrist, H.; Ternes, T.A.; von Gunten, U. Oxidation of pharmaceuticals during ozonation of municipal wastewater effluents: A pilot study. *Environ. Sci. Technol.* **2005**, *39*, 4290–4299, doi:10.1021/es048396s.
6. Elovitz, M.S.; von Gunten, U. Hydroxyl radical/ozone ratios during ozonation processes I: The R_{ct} concept. *Ozone Sci. Eng.* **1999**, *21*, 239–260. doi:10.1080/01919519908547239.
